# Rat-bite Fever: A Rare Diagnosis for a Common Pediatric Presentation: Case Report

**DOI:** 10.5811/cpcem.2021.5.51412

**Published:** 2021-10-05

**Authors:** Anthony Edholm, Marissa Heyer, Sondra M. Nemetski

**Affiliations:** * Hackensack University Medical Center, Department of Emergency Medicine, Hackensack, New Jersey; † Hackensack Meridian School of Medicine, Nutley, New Jersey; ‡ Joseph M. Sanzari Children’s Hospital, Hackensack Meridian Health, Division of Pediatric Emergency Medicine, Department of Emergency Medicine, Hackensack, New Jersey; § Hackensack Meridian School of Medicine, Department of Pediatrics, Nutley, New Jersey

**Keywords:** rat bite fever, rash, pediatric, zoonotic

## Abstract

**Introduction:**

Fever and rash is a common pediatric presentation to the emergency department but can present a diagnostic challenge to the clinician. Here we report the successful identification and treatment of a rare zoonotic exanthem that was facilitated by a thorough history and physical exam.

**Case Report:**

Rat-bite fever is a potentially fatal systemic illness characterized by relapsing fever, rash, and migratory polyarthralgias. Treatment includes antibiotics for *Streptobacillus moniliformis*, the most common pathogen, as well as appropriate hygiene education and prevention strategies. We report a case of *S. moniliformis* in the absence of an actual rodent bite.

**Conclusion:**

Due to the generally non-specific presentation of the illness, as well as the growing trend of caring for domestic rodents, it is crucial that clinicians ask details related to zoonotic and other exposures while obtaining medical histories.

## INTRODUCTION

Fever and rash are common reasons for children to seek medical care. While most pediatric exanthems are benign and self-limiting, there are several that represent true medical emergencies or demonstrate serious underlying causes. Identifying those cases can present a diagnostic challenge to the emergency clinician. We present a case of a young woman who experienced relapsing fever, petechial rash, and generalized arthralgias and myalgias, for whom a rare diagnosis was facilitated by a detailed history and physical exam.

## CASE REPORT

In May 2020 a well-appearing, immunized 11-year-old female, with a past medical history of depression, currently being treated with sertraline, arrived to the emergency department (ED) of a children’s hospital in the Northeastern United States with her mother for evaluation of a fever of 103.5°F (39.7°C), throbbing frontal headache, generalized muscle aches, and mild photophobia, all of which began three days prior to presentation. The patient also noticed that two days after these symptoms emerged, she developed a rash that started on her palms, distal wrists, and soles of her feet, and then spread proximally. Additionally, she had one episode of non-bloody, non-bilious vomiting after taking acetaminophen; however, she had since been able to tolerate food and fluids by mouth. The patient also endorsed phonophobia, mild sore throat, and neck pain with stiffness.

According to her mother, the patient was hospitalized for an episode of severe depression two months prior to this illness. After that episode, both the mother and patient thought that having a pet would improve the patient’s mental health. The patient reported acquiring four pet rats, which frequently crawled around her and occasionally nibbled on her skin; however, she denied ever having skin punctured. The patient’s mother reported that one of the rats was not vaccinated and had been sick; the animal was evaluated by a veterinarian and placed on antibiotics for an upper respiratory infection. The patient and her mother denied any contacts with ill humans; in fact, their social interactions in general had been limited, and the patient was attending school remotely, due to social distancing restrictions put into place during the coronavirus disease 2019 (COVID-19) pandemic.

**Image 1:**
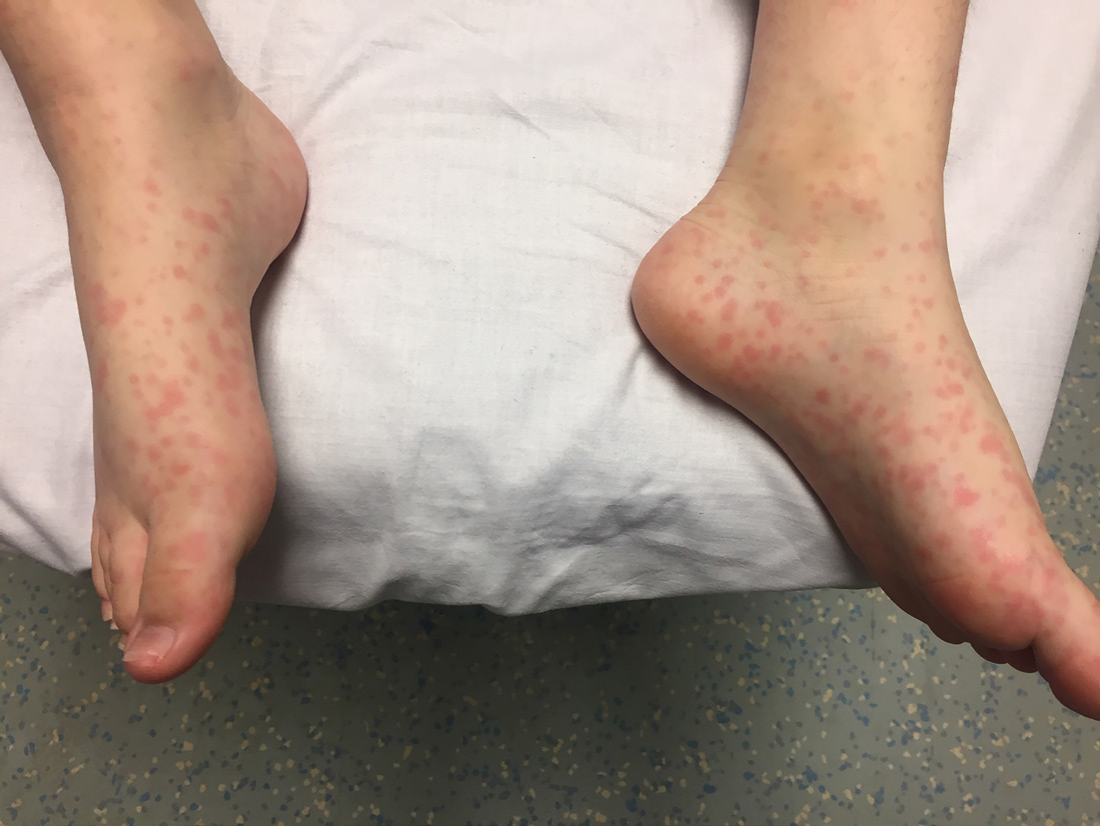
Erythematous maculopapular rash observed diffusely throughout the body and extremities with inclusion of soles.

**Image 2:**
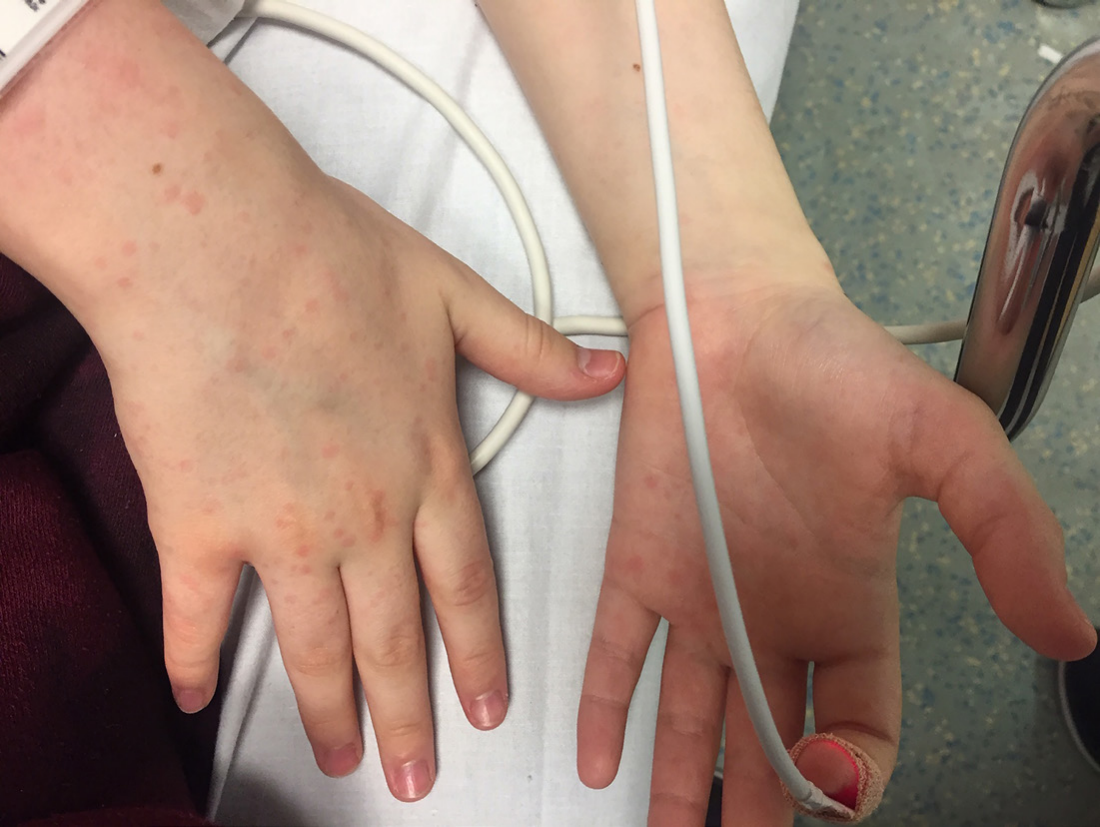
Erythematous maculopapular rash observed over palmar and dorsal surface of hands bilaterally.

On physical exam, the patient was alert and oriented with no signs of altered mental status or distress. Vital signs on presentation were significant for a blood pressure of 105 per 52 millimeters mercury, pulse rate of 111 beats/minute, and temperature of 99.9°F (37.7°C). Examination revealed full range of motion of the neck, and both Kernig and Brudzinski signs were negative. The patient had minimal tenderness to palpation of the suprapubic abdominal region and reported myalgias with pain upon palpation of the skin, legs, knees, and elbows. A diffuse, blanching, erythematous, macular rash was observed on bilateral palms and soles (Images 1 and 2), extremities, trunk, and face. Pulmonary and cardiac exams were normal.

Given the patient’s history and physical exam findings, the infectious disease team was consulted for a suspected diagnosis of rat-bite fever (RBF). Differential diagnoses also included strep pharyngitis and viral exanthem. Meningitis, Rocky Mountain spotted fever, ehrlichiosis, and severe acute respiratory syndrome coronavirus 2 (SARS-CoV-2) were less likely due to the patient’s clinical presentation and no known exposures. Labs, urinalysis, and blood cultures were ordered. A lumbar puncture was considered pending results; however, the patient remained hemodynamically stable and non-toxic appearing, so the procedure was ultimately deferred. Group A streptococcus titer results were negative. Urinalysis was positive for moderate bacteria and white blood cells, but suspicion for urinary tract infection was low since the patient did not have any urinary complaints. The patient was monitored and started on intravenous (IV) fluids and broad-spectrum antibiotics.

**CPC-EM Capsule**

What do we already know about this clinical entity?

*Rat-bite fever is a rare systemic illness characterized by relapsing fever, rash, and migratory polyarthralgias, with a high mortality rate if not treated promptly.*

What makes this presentation of disease reportable?

*Despite the lack of a rodent bite, the case was successfully identified and treated based on a thorough history and physical exam.*

What is the major learning point?

*Due to the wide range of differential diagnoses, it is critical to rely on a systematic approach toward analyzing patients presenting with fever and rash.*

How might this improve emergency medicine practice?

*The approach presented here will aid in distinguishing between benign and emergent causes of pediatric rashes.*

Since the case occurred during the height of our hospital system’s COVID-19 surge, and available beds were limited, the patient’s mother was reluctant to agree to admission for IV penicillin and close monitoring. After discussing the case with the on-call pediatric infectious disease physician and shared decision-making with the patient and her mother, the patient was given a dose of ceftriaxone in the ED and discharged on amoxicillin 500 milligrams three times daily. The patient’s mother assured close follow-up with the patient’s pediatrician within 48 hours of discharge and was advised to return to the ED for any worsening of symptoms. Upon follow-up, the patient’s mother reported resolution of fever within two days and rash within three days of the ED visit.

## DISCUSSION

Rat-bite fever is considered a rare disease but is not nationally reportable, and many cases may go unreported as a result of failure to properly diagnose.^1^ In the Western hemisphere, RBF is typically caused by Streptobacillus moniliformis, a pleomorphic, Gram-negative, facultative anaerobe that inhabits the oropharynx of primarily rats and other domestic rodents.^2^ S moniliformis requires microaerophilic conditions to grow; as a result, detection by conventional microbiological techniques is quite difficult and may delay correct and prompt diagnosis and treatment.^3^,^4^

The natural reservoir responsible for transmitting *S. moniliformis* is rats. The bacterium classically colonizes the upper respiratory tract of the rodent,^5^ and while most rats are asymptomatic, some may occasionally show signs and symptoms of an upper respiratory tract infection. Historically, individuals at high-risk for developing RBF are those in occupations that involve direct handling of rats, including laboratory workers, pet store employees, and veterinary staff. However, the incidence of RBF is believed to have increased in recent years, particularly in young children – who represent over 50% of cases – as a result of greater pet rat exposure.^2^,^6^ While the precise rate of rat and other rodent ownership is unknown, the 2019–2020 American Pet Products Association’s National Pet Owners Survey found that an estimated 5.4 million American households own a “small animal” as a pet.^7^ Additionally, the American Veterinary Medical Association reported that in 2016 2.6% of United States households owned ferrets or “other mammals,” exclusive of dogs, cats, or rabbits.^8^ Therefore, obtaining a thorough history of animal exposure, as well as animal milk sources, is crucial when suspicion of a zoonotic illness is high.^5^

As its name suggests, RBF can be transmitted directly via a bite or scratch from rodents, including rats, mice, and possibly gerbils, guinea pigs, and ferrets, infected with the bacteria by entry through a wound, open skin, or mucous membranes.^1^ Additionally, the zoonotic bacteria can be transmitted through contact with the urine, saliva, or droppings of infected rats, through contaminated surfaces, or via consumption of food or drinks contaminated with urine or droppings of an infected rodent.^9^ However, up to a third of patients do not report a known bite or rodent exposure.^1^,^10^

On average, clinical symptoms develop 3–10 days after initial exposure, but may be delayed up to three weeks later.^3^ When illness occurs, patients commonly present with abrupt onset of fever and rigors; the fever may resolve within a few days but ultimately relapses.^3^ Additional frequently reported symptoms include headache, vomiting, migratory polyarthralgias of large and small joints, and rash.^11^ The rash may appear as maculopapular, petechial, or purpuric, and involve the extremities, particularly the hands and feet.^12^ In some cases, exquisitely tender hemorrhagic vesicles may develop which, in the setting of an otherwise nonspecific febrile illness, are strongly suggestive of infection with *S. moniliformis*.^3^,^11^ Although rare, complications of untreated RBF include abscess formation, hepatitis, nephritis, polyarteritis nodosa, meningitis, pneumonia, pericarditis, myocarditis, and endocarditis.^13^ While RBF usually responds quickly to penicillin, the mortality rate of untreated RBF is approximately 10%.^3^

Penicillin is still regarded as the first-line treatment for proven or highly suspected cases of RBF. Treatment is generally given intravenously for the first 5–7 days, and then orally for an additional seven days.^1^,^6^ Streptomycin, tetracycline, and cephalosporins are recommended for penicillin-allergic patients.^1^ However, because tetracycline is known to cause dental abnormalities in children, it should not be used as a first-line agent in pediatric patients.

Proper hygiene education and strategies for prevention of future infections is very important, especially in children and adolescents with pet rats. Emphasis should be placed on safe play to reduce the transmission of germs and reduce the risk of bites from the rodent, as well as proper cleansing and disinfection of hands, surfaces, and rodent habitats and supplies.^11^

Pediatric presentation of fever, migratory polyarthritis, and rash evokes an extensive differential diagnosis. Potential bacterial causes include *Streptococcus pyogenes* (and associated diseases), *Staphylococcus aureus*, disseminated gonorrhea, and meningococcemia. Other zoonotic illnesses, such as Lyme disease, ehrlichiosis, brucellosis, and rickettsial infections, particularly Rocky Mountain spotted fever, should be thoroughly explored during history taking.^3^ Viral etiologies, such as parvovirus B19, coxsackievirus, and reactive arthritis secondary to viral illness, as well as inflammatory considerations including juvenile idiopathic arthritis, should also be considered.^4^

When evaluating a patient, demographics, season, and geography should be taken into consideration. Our patient was an 11-year-old female being evaluated in an urban region of New Jersey in mid-May. History taking should focus on the onset, progression, associated symptoms, and attempted therapies thus far. In addition, the physician must consider the patient’s past medical history, prescribed medications, use of recreational substance or herbal supplements, vaccination status, and allergies. Social elements are often critical in determining rare diagnoses; therefore, the ED evaluation should include discussions concerning recent activities, occupations, typical diet, living situation, animal exposures, sexual activity, recent travel, and hobbies.

Next, emphasis should be placed on performing a detailed physical exam, which should broadly include features of the rash, such as petechial or purpuric lesions, erythematous appearance, maculopapular appearance, or vesiculobullous development. Extra care should be taken to identify potential dermatological emergencies, which can be suggested by associated systemic symptoms as well as specific features of the rash, including lack of blanching; evidence of crepitus; involvement of the palms, soles, genitalia, or mucous membranes; warmth or tenderness to palpation; and presence of Nikolsky’s sign.

The patient presented here endorsed specific clues identified on social history and findings on physical exam that helped identify the underlying cause of her symptoms. First, she was found to have prolonged rodent exposure from a pet source. Second, the rash demonstrated unusual features, including involvement of her palms and soles. An accurate travel history, coupled with consideration of the season and geography, also helped exclude alternative diagnoses including Rocky Mountain spotted fever. Knowledge of her complete vaccination status, recent social interactions (ie, minimal exposure to other children due to social distancing measures during the COVID-19 pandemic), and a thorough exam allowed appropriate ranking of other infectious causes within the differential diagnosis. This comprehensive approach to the history and physical exam provided the information needed to make the diagnosis with confidence, even without laboratory confirmation.

Safe discharges from the ED are multifactorial. Clinicians should consider discharge if the interventions initiated in the ED have corrected the course of the patient’s pathology, any residual symptoms are tolerable and can be managed with outpatient interventions, the patient does not demonstrate altered mental status, the patient is able to reiterate the shared plan of care back to the provider in their own words, vital signs are within normal limits, and appropriate, reliable follow-up is assured. Patient autonomy and shared decision-making regarding the risks and benefits of admission vs discharge should also weigh upon disposition decisions, especially in borderline or complex cases.

In the case reported here, once the underlying pathology was identified, interventions such as IV antibiotics, analgesia, and antipyretics were initiated to treat the patient’s underlying pathology and associated symptoms. While in most cases, RBF is treated with multiple doses of IV antibiotics in the hospital, in this instance the risk of nosocomial infection during the height of the COVID-19 pandemic, as well as the limited availability of inpatient resources at the time, complicated what would usually have been a straightforward disposition. Shared decision-making was then made regarding disposition between the patient, her mother, the infectious disease physician, and the ED team. The patient appeared alert and oriented to person, place, and time, with no signs of altered mental status. She endorsed significant symptomatic improvement following interventions and was in no distress with stable vital signs. Furthermore, the patient and her mother demonstrated understanding of the pathology of the disease, the antibiotic course required to treat it, and the need for close follow-up and continued monitoring of symptoms, as well as signs or symptoms that would warrant prompt return to medical care. Our team agreed to follow up with the patient’s mother over telephone within 24 hours and she would follow up with her primary care provider within three days. Careful considerations of all these factors allowed for a safe discharge and a positive outcome for this patient.

## CONCLUSION

Rat-bite fever is a potentially fatal illness characterized by systemic symptoms, particularly relapsing fever, rash, and migratory polyarthralgias. While considered rare and non-reportable, over 50% of cases have been recorded in children, potentially due to the growing popularity of keeping rats as pets.^1^.^2^ Due to the high risk of mortality if left untreated and the generally non-specific presentation, it is important for physicians to take a thorough medical history, taking particular note of potential exposure to animals, both wild and domesticated, when treating patients with potential zoonotic illnesses.^14^ Furthermore, a comprehensive and systematic approach to the history and physical exam is essential in distinguishing between benign and emergent causes of pediatric rashes.
